# How Crucial is the Functional Pit Organ for the *Varroa* Mite?

**DOI:** 10.3390/insects11060395

**Published:** 2020-06-26

**Authors:** Beatrice T. Nganso, Kannan Mani, Yam Altman, Ada Rafaeli, Victoria Soroker

**Affiliations:** 1 Institute of Plant Protection, Agricultural Research Organization, The Volcani Center, P.O.B 15159, Rishon leZion 7505101, Israel; beatricen@volcani.agri.gov.il (B.T.N.); ahilkannanbdu@gmail.com (K.M.); yamalt88@gmail.com (Y.A.); vtada@volcani.agri.gov.il (A.R.); 2 Mina and Aberhard Gudman Faculty of Life Sciences, Bar-Ilan University, Ramat Gan 5290002, Israel

**Keywords:** honey bees, Acari, reproduction, parasite–host interaction

## Abstract

Olfaction as well as gustation, are essential for animal survival, allowing behavioral modulation according to environmental input. We focused our study on an obligate ecto-parasitic mite of honey bees, the *Varroa destructor* Anderson and Trueman (Parasitiformes, Mesostigmata, Varroidae). By mechanically blocking the main olfactory organ on *Varroa* forelegs by varnishing with nail polish, we were able to show that other sensory organs cannot significantly compensate chemosensory abilities required for mite’s host selection, identification as well as reproduction. In fact, we found that mites with blocked forelegs had a significantly lower ability to reach a host bee than those with varnished idiosoma and unvarnished control. Furthermore, fewer foreleg blocked mites were feeding on the nurse bees and their reproduction in the brood cells was significantly impaired. The inhibition of reproduction was also reflected in altered expression levels of vitellogenin and vitellogenin receptor genes in foreleg-blocked mites.

## 1. Introduction

Chemosensing is the primary and apparently the oldest mode of sensing in nature. Olfaction as well as gustation are essential for animal survival, allowing behavioral modulation according to environmental input thus optimizing detection of food, mates and enemy-avoidance via volatile and contact chemicals. As the most successful group of animals on earth, arthropods possess remarkable abilities of chemosensing. Their olfactory neurons are encased in sensory hairs, mostly located on appendages such as the antenna in insects [1] or the forelegs in Acari [2]. In addition, gustatory receptors are often associated with the mouthparts and tarsi [3].

We focused our study on the mite, *Varroa destructor* Anderson and Trueman (Parasitiformes, Mesostigmata, Varroidae). This mite is an obligate ecto-parasite of honey bees (*Apis* spp.) and is considered as the major driver of honey bee colony losses almost worldwide [4]. The mite reproduces and develops within the honey bee brood cell and its life cycle is well synchronized with that of its host, the honey bee. *Varroa*’s life cycle can be generally divided into two main phases: a non-reproductive, also called phoretic phase, in which the female mite parasitizes an adult bee (nurse or forager) and a reproductive phase, in which the mite parasites the honeybee pupae and reproduces within the sealed brood cell [5]. During the non-reproductive phase, the female mite can be carried by a forager bee to another hive, by a nurse bee to a brood cell or move freely on the surface of the combs. The entrance of the female mite into the brood cell is synchronized with the developmental stage of the larvae, and occurs shortly before the cell is sealed [6].

The strong association between the mite’s and bee’s life cycles underscores the importance of understanding chemical perception in the *Varroa* mite, which is essential for its host finding, selection and reproduction [5,7]. Recently, we found that the ability of the mite to select and sense bee volatiles are apparently stage-dependent; and even though both reproductive and non-reproductive mites usually exhibit the same host preference, the non-reproductive mites are more successful in reaching a host than reproducing ones [8]. Still, apparently reproductive mites are also sensitive to host odors as their reproduction is activated or switched off by cues coming from an appropriate bee brood [9].

Information on host searching behavior by the mite and the nature of chemical cues from the host bee and their environment that regulate *Varroa*’s reproduction suggests that highly volatile and low volatile cues are perceived by the mite; thus implicating the involvement of both olfactory and gustatory sensory receptors, respectively [10,11]. These sensory receptors are mainly localized in the forelegs and gnathosoma [12]. In fact, while walking, the mite usually raises its forelegs above the ground in a similar manner to the antennae of other arthropods thereby suggesting that the forelegs may be primarily used for volatile detection. The olfactory organ located on the distal dorsal part of each foreleg is analogous to Haller’s organ found in ticks, and consists of nine chemosensory sensillae located inside a pit, and nine more sensillae surrounding it [13]. At least one of the pit sensillae and four among the surrounding sensillae were suspected by their morphology to act in gustation [13]. Previously, we demonstrated that isolated *Varroa* forelegs respond electro-physiologically to both nurse and forager honey bee volatiles and honey bee pheromone, E-β-ocimene [14]. Moreover, we shown that disruption of foreleg volatile chemo-sensing by specific dialcoxybenzenes, cyclopentenol ethers and N,N-Diethyl-m-toulamide (DEET) is reflected in changes in host preference and detection abilities by free moving mites [14,15]. Within the gnathosoma, gustatory tip-pore sensilla in the pedipalps and the chelicerae are well known in ticks [16]. In *Varroa* mite, the pedipalps consist of four segments each covered by strong trichoid setae and in addition, each distal segment of the pedipalps contains long setae [13]. It was reported that some of the pedipalpi setae are tip pore sensillae [12], however the significance of these in host detection and recognition is still unknown. Additionally, it remains unknown if additional body parts of *Varroa* contain chemosensory sensillae involved in host chemosensing.

In this study, we aimed to evaluate the relative contribution of *Varroa*’s sensory organs on the forelegs and gnathosoma, to host sensing and selection as well as their reproduction.

## 2. Materials and Methods

### 2.1. Biological Material

Honey bee colonies (*A. mellifera liguistica*) were maintained at an experimental apiary at the Agricultural Research Organization (ARO), Volcani Center, Israel. The experimental hives were maintained on netted bottom boards without any treatment against *Varroa*. Non-reproductive mites were collected by sugar shake method from three unrelated highly infested honeybee colonies while three honeybee colonies with a low level of infestation were used for artificial infestation. We used only active mites in all the experiments. Worker honeybees that were observed with their heads in brood cells for at least 2–3 s were collected as nurse bees whereas those that landed at the entrance of the hives with a pollen load were collected as pollen foragers as described by Eliash et al. [15].

### 2.2. Manipulation of Female Mites for Bioassays

**Figure 1:**
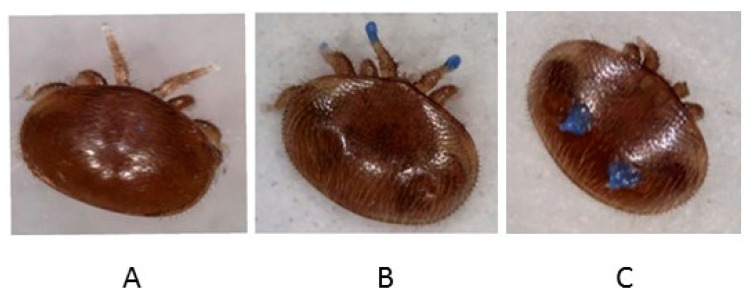
*Varroa destructor* used for the experiments: unvarnished female mite (**A**), mites with varnished foreleg (**B**) and mites with varnished idiosoma (**C**).

We manipulated the mite’s olfactory perception by preventing them from perceiving volatiles from the honeybees using their forelegs such that they will need to rely on other organs for chemo-perception. To do so, we varnished their forelegs with Majorelle Blue nail polish (No.495, GA-DE Cosmetics, Israel) and compared their behaviors with unvarnished mites according to Häußermann et al. [17] (Figure 1A,B). We also evaluated a possible negative effect of the commercial nail polish by varnishing another group of mites with this nail polish on the dorsal side of their idiosoma only and compared their behaviors with unvarnished mites (Figure 1C). In both scenarios, we evaluated the impact of foreleg blocking on host sensing, orientation and/or detection as well as mite reproduction in the assays described below. The varnished mites were used three hours post-painting in all the experiments.

#### 2.2.1. Host Orientation Behavior

It is well known that *Varroa* mites prefer nurse to forager bees to improve their reproductive fitness in honey bee colonies [5]. Therefore, we examined the mites’ ability to select between a nurse bee and a pollen forager as well as the rate at which they reached any host using a two-choice bioassay in glass Petri dishes (100 mm diameter) described by Singh et al. [14] and Eliash et al. [15]. Both nurse and forager bees were freshly collected, frozen for 2 h and subsequently tempered before the trial for approximately 30 min to about 30 °C. Briefly, a single mite was released in the center of a wet filter paper placed in a Petri dish and was allowed to choose between freshly killed bees (a nurse bee and a pollen forager (after pollen removal)), placed at opposite sides. The dishes were maintained closed throughout the entire experiment. We kept the trays with plates on different angels on the bioassay table during the trial to avoid positional bias. The position of the mite on a host bee or elsewhere was noted after 1, 2, 3 and 24 h. A total of twenty mites were tested for each treatment category. Mites and honey bees were sourced from different colonies. The bioassays were conducted in a dark room at 30 °C and 50–60% R.H. The light was turned on briefly during each observation time to record the position of the mites. At the end of the bioassay, we calculated the mite’s host preference and its ability to reach any of the hosts as the percentage of mites reaching a nurse bee or a pollen forager, or any of the host out of the total mites tested that remained viable at the end of the experiment.

#### 2.2.2. Host Identification and Feeding

Recently, it was demonstrated that *Varroa* mites are usually found feeding on the honey bee’s fat body on the ventral side of the third abdominal segment underneath the sternite or tergite [18]. Therefore, using a non-choice bioassay, we examined the ability of the mites (with varnished and unvarnished forelegs) to recognize a freshly killed nurse bee as a suitable host and to locate their feeding site on the host. Briefly, a nurse bee was placed in the center of a wet filter paper contained in a glass Petri dish as described above. We placed a single mite on the nurse bee, precisely on the 7th abdominal segment on the ventral side to determine if it recognized and stayed on the host or walked away. Mites, which stayed on the nurse bee were further assessed as in Ramsey et al. [18] to determine whether they were feeding. Thirty-nine and forty mites with unvarnished and varnished forelegs were tested, respectively. The positions of the mites on the bee and on the feeding site or elsewhere were noted after every half an hour for up to 3 h. The percentage of mites that stayed on the bees out of the total mites tested that remained viable at the end of the bioassay was calculated. We also calculated the percentage of mites that were found feeding out of the total live mites that stayed on the bees.

#### 2.2.3. Mite Reproduction

We manually infested newly capped worker brood (within 6 h post capping) with one mite from one of the three treatment groups: unvarnished mites, mites with varnished idiosoma and mites with varnished forelegs as described by Dietemman et al. [19]. The combs were returned to their original colonies following infestation. After 72 h of incubation, the cells were uncapped and the mite reproduction was assessed in each cell as well as the condition of the bee pupae and the mites. Particularly, the number of mites dead, alive with white patch (indicating defecation and thus feeding) and mites with white patch and egg (indicating reproduction) were recorded. The number of cleaned brood cells were also recorded. At the end of the experiment, we calculated the number of alive and dead mites from the total number of mites tested in each treatment group and which were found in brood cells that were not cleaned by the bees. From the total number of mites that remained alive in each treatment group, we further calculated the number of reproductive and non-reproductive mites. All the live mites were subsequently collected and stored at −80 °C for further analysis of the expression levels of vitellogenin 2 (Vg2) and vitellogenin receptor (VgR) genes by real-time quantitative polymerase chain reaction (RT-qPCR). Another group of phoretic mites with varnished and untreated forelegs were collected and the expression of these genes was also determined in those mites considered as control.

Total RNA was extracted from each group of mites using the GeneAll Kit (Seoul, South Korea) as previously described by Singh et al. [8]. Three to five biological replicates were used for each treatment and 5 mites were used per replicate. The quality and quantity of the RNA samples were measured using a NanoDrop 2000 spectrophotometer (Thermo Scientific, Wilmington, DE, USA). Purified RNA (400 ng) was used for cDNA synthesis using the qPCRBIO cDNA Synthesis Kit (Thermo Scientific, Waltham, MA, USA) following the manufacturer’s recommendations. Sets of primers for Vg2 gene were used as reported by Cabrera Cordon et al. [20] (Table S1). Sets of primers for *Varroa*’s putative VgR gene (GenBank accession: XM_022814228.1) were designed using the NCBI primer design tool (Table S1). These primers were designed from the open reading frame (ORF) that contained the conserved domain of the gene according to hmmscan (HmmerWeb version 2.7.2 (http://hmmer.org/) [21]. As a normalizing gene, we used the *Varroa*’s 18S ribosomal RNA (18S) gene [22] (Table S1). Amplifications were performed using qPCRBIO SyGreen Blue Mix Hi-Rox (Thermo Scientific, Waltham, MA, USA) in Rotor Gene (RG)-3000 (Corbett Robotics Pty, Brisbane, Australia) with RG-6000 series software 1.7 for analysis of gene expression levels. The cycling conditions were as follows: 15 min activation at 95 °C, 40 cycles of 15 s at 95 °C, 30 s at 57 °C and 30 s at 72 °C. A melting ramp from 72 to 95 °C was used with a 1 °C rise at each step and a 5-s interval between steps. Standard curves for each set of primers were evaluated to ensure that they amplified the target genes efficiently. For all qPCR assays, a no-template control was included (data not shown). The expression levels of Vg2 and VgR genes were calculated after normalization with 18S using the Delta Delta Ct Relative method [23].

### 2.3. Data Analyses

The analyses were mostly performed using JMP^®^, 14, SAS Institute Inc., Cary, NC, 1989–2019. We also used the Chi-square test function in R to compare the proportion of mites that reached a nurse bee or a pollen forager in each treatment category: unvarnished mites, mites with varnished idiosoma and forelegs at final time point (24 h). We used Nominal Logistic Fit model in JMP^®^ to compare the proportion of mites that stayed or fed on the nurse bee between mites with varnished and unvarnished forelegs over time. For the reproductive data, the Chi-square test was also used to compare the proportion of alive, dead, reproductive and non-reproductive mites among the treatment groups. The differences in expression levels of Vg2 and VgR genes among the treatment groups were analyzed using a one-way ANOVA followed by a post hoc Tukey–Kramer test.

## 3. Results

### 3.1. Host Orientation Behavior

**Figure 2:**
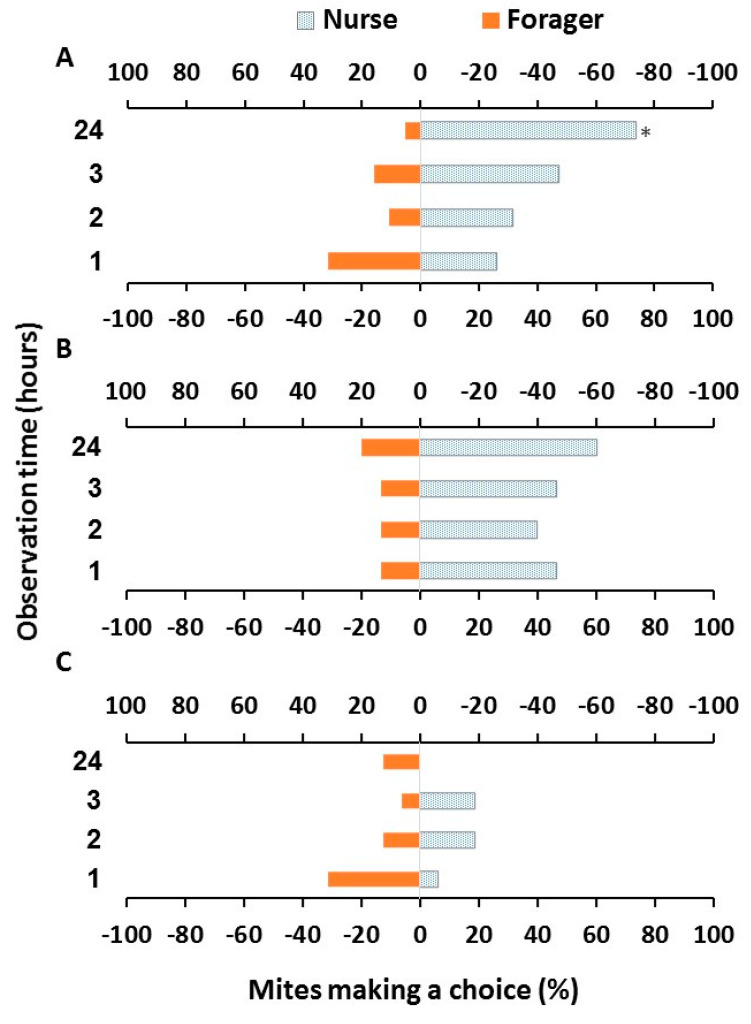
Host selection (%) of surviving mites at the end of the experiment, for unvarnished mites, *n* = 19 (**A**), mites with varnished idiosoma, *n* = 15 (**B**) and mites with varnished forelegs, *n* = 16 (**C**) at 1, 2, 3 and 24 h. Bars with asterisks (*) indicate significant difference in host selection between a nurse and a pollen forager bee (Chi-Square test, *p* < 0.05).

Most of the mites (75–95%) survived until the end of experiment. No significant difference in mite survival was found among treatments (Chi-Square = 3.62, df = 2, *p* = 0.164). When comparing the behavior of mites from the three groups over the four time points we found a significant effect of treatment (Chi-Square = 8.6522, df = 2, *p* = 0.0132) but no significant effect of time (Chi-Square = 2.2687, df = 3, *p* = 0.5185) or interaction between the time and treatment (Chi-Square = 9.4559, df = 6, *p* = 0.1495). In fact, unvarnished mites generally preferred a nurse to a forager bee. The choice was significant after 24 h (Chi-Square = 15.86, df = 1, *p* < 0.05) (Figure 2A). Similarly, mites with varnished idiosoma preferred a nurse to a forager bee, though this was almost significant after 24 h (Chi-Square = 3.47, df = 1, *p* = 0.06) (Figure 2B). When just the behavior of the untreated and idiosoma varnished mites was compared, no effects of treatment (Chi-Square = 0.1254, df = 1, *p* = 0.9392), time (Chi-Square = 0.0405, df = 3, *p* = 0.9979), or interaction between time and treatment (Chi-Square = 3.9845, df = 6, *p* = 0.2631), were found. In contrast, unlike mites of these two groups, mites with varnished forelegs did not show significant selection ability even after 24 h (Chi-Square = 0.53, df = 1, *p* > 0.05) (Figure 2C).

**Figure 3:**
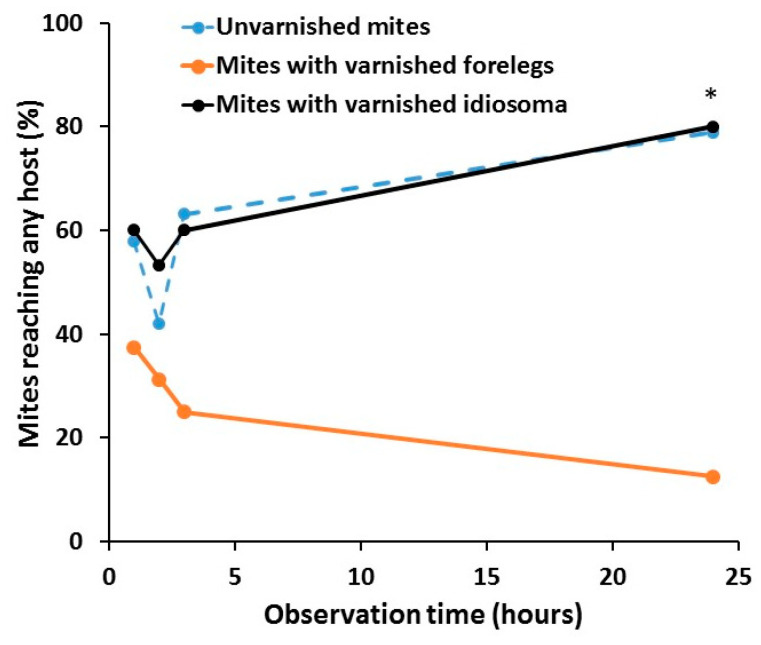
Ability of unvarnished control mites (*n* = 19), mites with varnished idiosoma (*n* = 15) and mites with varnished forelegs (*n* = 19) to reach any of the host bee after 1, 2, 3 and 24 h. The data are percentages of all mites that remained alive (out of 20 mites tested in each group) at the end of the experiment. Asterisk (*) indicates significant difference in the ability to reach any host among the treatment group (Chi-square test, *p* < 0.05).

The mite’s ability to reach any of the host bee was affected significantly by treatment (Chi-Square = 23.6975, df = 2, *p* < 0.001) but not by the time (Chi-Square = 1.6978, df = 3, *p* = 0.6414) or interaction between the time and treatment (Chi-Square = 8.4555, df = 6, *p* = 0.2066). At all times points, a lower number of mites with varnished forelegs reached any bee than those from the other two groups of varnished idiosoma and unvarnished control. When just the behavior of the untreated and idiosoma varnished mites was compared, no effects of treatment (Chi-Square = 0.0999, df = 1, *p* = 0.7519), time (Chi-Square = 7.6610, df = 3, *p* = 0.0536), or interaction (Chi-Square = 0.3633, df = 6, *p* = 0.9479), were found (Figure 3).

### 3.2. Host Identification and Feeding

**Figure 4:**
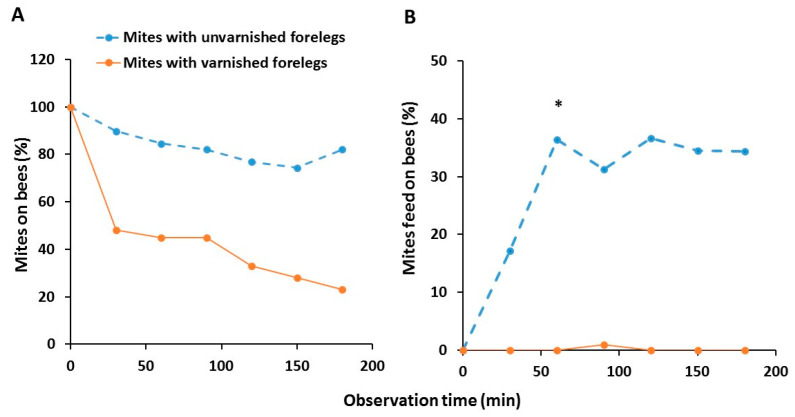
Ability of mites with unvarnished (*n* = 39) and varnished forelegs (*n* = 40) to recognize nurse bees as suitable hosts (**A**) and feed on them (**B**) after every half of hour up to 3 h. The data are percentages of all mites that remained alive until the end of the experiment. Asterisk (*) indicates significant difference in the ability to reach any host among the treatment group (Chi-Square, *p* < 0.05).

None of the mites died during this experiment. The ability of the mites to recognize and stay on the nurse bee was significantly affected by both time (Chi-Square = 11.15, df = 5, *p* = 0.0484) and the treatment (Chi-Square = 104.39, df = 1, *p* < 0.0001). Significantly less mites with varnished forelegs stayed on the host than those with unvarnished forelegs (Figure 4A). There was no significant interaction between the time and treatment (Chi-Square = 2.35, df = 5, *p* = 0.7987). Even though not all mites that stayed on the bee were feeding, significantly fewer mites with varnished forelegs were found feeding on the nurse bees than unvarnished mites, specifically on the ventral side of the third abdominal segment underneath the sternite. This difference was significant after 1 h (Chi-Square = 6.66, df = 1, *p* < 0.05) (Figure 4B). Overall, there was a significant difference between the groups (Chi-Square = 44.05, df = 1, *p* < 0.0001), but there was no effect of time (Chi-Square = 3.34, df = 5, *p* = 0.6480) and the interaction between the treatment and time was also not significant (Chi-Square = 3.07, df = 5, *p* = 0.6886).

### 3.3. Mite Reproduction

**Figure 5:**
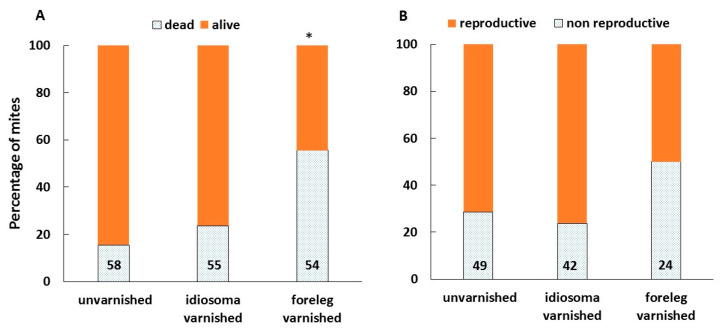
The effect of treatments on the rate of survival (**A**) and reproduction of mites (**B**) in artificially infested brood cells. Numbers at the bottom of bars indicate the total number of mites for each treatment. *—indicates bar that is significantly different from the other two, Chi-Square, *p* < 0.0001.

Following a 72-h incubation, 20% of the artificially infested brood was gone, apparently removed by hygienic workers. The percentage of brood removed was not significantly different between the treatments (Chi-Square = 0.78, df = 2, *p* = 0.68). However, the condition of the remaining mites differed between the treatments. The mortality rate was significantly higher in mites with varnished forelegs than the unvarnished and idiosoma-varnished mites (Chi-Square = 22.74, df = 2, *p* < 0.0001) (Figure 5A). Among the mites that remained alive and fed (as was noticed by the presence of the white patch of secretions on the cell wall), fewer mites with varnished forelegs reproduced than the unvarnished and idiosoma-varnished mites, though this difference was only close to significance (Chi-Square = 4.941, df = 2, *p* = 0.084) (Figure 5B).

**Figure 6:**
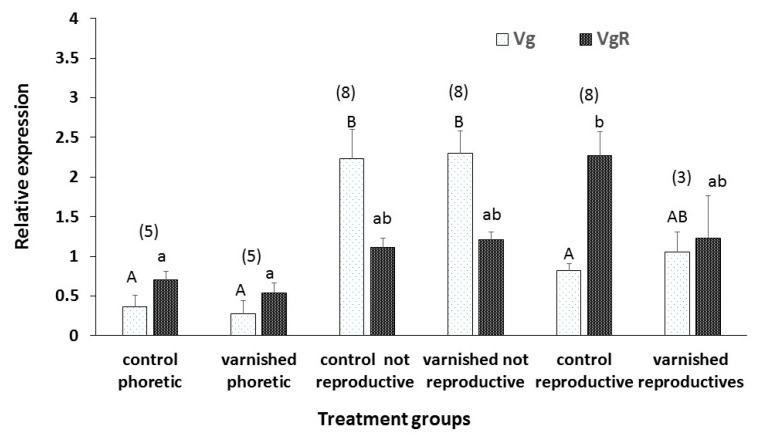
Relative expression of vitellogenin (Vg) and vitellogenin receptor (VgR) in phoretic mites with varnished and unvarnished forelegs as well as four groups of mites collected from worker brood cells after 72 h of infestation: control and varnished mites that were alive but not reproduced and control and varnished mites that had laid an egg. Bars indicate mean ± s.e. Numbers above bars in brackets indicate the number of replicates in each treatment used in RT-qPCR analysis. Different letters above bars indicate treatments that differ significantly, within each transcript, when compared using ANOVA, followed by Tukey HSD, *p* < 0.05.

Expression pattern of vitellogenin (Vg) and VgR genes between the treatments/physiological stages is shown in Figure 6. The expression level of Vg2 was highest in the non-reproducing varnished and control mites after 72 h post infestation in the cells with worker pupae, than in the other treatment groups (ANOVA: F_(5,31)_ = 10.17, *p* < 0.0001). In fact, the lowest level of Vg2 was found in phoretic mites with varnished and unvarnished forelegs as well as in the reproductive control mites whereas the level of this gene in forelegs of varnished reproducing mites was intermediate. Contrary to the expression level of Vg2, the expression level of VgR was highest in the reproducing control mites than in the other treatment groups (F_(5,31)_ = 3.66, *p* = 0.01). In fact, the VgR level was lowest in phoretic mites with varnished and unvarnished forelegs whereas its levels in non-reproducing mites from the two groups and reproducing forelegs varnished mites were similar and intermediate, respectively.

## 4. Discussion

Although the role of the pit sensory organ located on the forelegs of female *Varroa* mites in host chemosensing was known already for some time [13,24], the significance of the other sensory organs on the mite’s gnathosoma or other body parts remains unknown. In this study, by blocking the tarsal pit sensory organ located on the female’ s forelegs with a commercial nail polish we were able for the first time to demonstrate the crucial role of the this organ in host orientation, selection and reproductive behaviors. We found that control mites with unvarnished sensory organs exhibited the usual preference for honeybee nurse over foragers [5,10,11], which increased with time. The same was true for mites with varnished idiosoma, suggesting that the commercial nail polish used in our study had no negative effects on *Varroa*’s behavior towards its host. In contrast, the mites with varnished forelegs were unable to maintain the common preference of nurse over forager honeybees over time. Additionally, their ability to reach any host was drastically impaired. Direct observation of mites’ behavior indicates that foreleg varnished mites move differently from foreleg untreated mites. They lifted their forelegs in the air but unlike untreated mites the foreleg blocked mites mostly did not move (S1 movie). If they eventually reached the bee they did not attach between its abdominal segments for feeding but after staying for some time on the body parts such as wings or thorax, without feeding, they mostly moved off the bee thus indicating that their host sensing was severely impaired. Taken together, these data provide additional evidence for the biological significance of *Varroa*’s tarsal pit sensory organ on the forelegs for host chemosensation [14,15].

In this study, we anticipated that mechanical blocking of the pit sensory organ on *Varroa*’s forelegs would only partially affect their host acceptance at short range due to chemosensillae presumably present on the gnathosoma as was reported by Liu and Peng [12]. However, approximately 50% of mites with varnished forelegs left the nurse bees within the first half an hour of the bioassay and only about 20% remained after 3 h. Moreover, we found that practically none of the forelegs-varnished mites were feeding on the bee during the entire observation period whilst about 32% of the control mites fed on the bee. The low acceptance by the control group probably resulted from the fact that in our bioassay we did not use alive bees but freshly killed ones. As additional cues such as humidity and carbon dioxide may mediate the interaction between the female mite and its environment, these data cannot completely rule out the possibility that sensillae of the gnathosoma have chemosensory function. However, the data obtained herein indicated that the pit organ located on the forelegs is without a doubt the main sensory organ in host orientation, selection and acceptance in the non-reproductive mites.

As chemical cues are important not only for the mite’s host finding and selection but also for the induction of its reproduction [20], we questioned the role of sensory organs on the forelegs and gnathosoma during mite reproduction. During this stage, *Varroa* is very sensitive to the cues from the prepupae. These cues are composed of aliphatic alcohols and aldehydes, eicosane, esters as well as C_19_ to C_29_ hydrocarbons [5,9,10,11]. In particular, changes in the relative ratio between fatty acid methyl and ethyl esters were reported to trigger the first steps of mites’ oogenesis [9]. We thus anticipated that if blocking the pit organ disrupts host chemosensing it will subsequently prevent induction of oogenesis as will be reflected in a change in transcription of vitellogenin and vitellogenin receptor and eventually in reproduction itself. Our results demonstrate even more dramatic effect on mite survival, reproduction and gene expression. In fact, varnishing mites’ forelegs prior to introduction to the prepupal cell had a dramatic impact on mite’s survival. Fifty-six percent died and only half of the surviving forelegs-varnished mites reproduced. While 84% of control and 76% of the idiosoma-varnished mites survived and more than 70% reproduced. The high mortality of the forelegs-varnished mites could be a result of the inability of the mites to recognize and feed on the host as was shown above in the non-reproductive mites. Survival of the remaining foreleg-varnished mites could be a result of compensation by other sensory organs or incomplete block of the sensory organs by the nail polish or self-removal of nail polish from sensory organs. It is worth mentioning that at least in some of the forelegs varnished mites, the varnish appeared partially removed by the end of 72 h of incubation.

The fact that some of the control and idiosoma-varnished mites did not reproduce within 72 h post infestation is not surprising as this phenomenon was also reported in previous studies (reviewed in [5]). Non-reproduction of mites in these two treatment groups could indicate merely a delay in egg laying. However, it is obvious that the process of egg development had started as could be judged by the significantly high levels of vitellogenin (Vg) and VgR genes. Of note, Vg is the precursor of vitellin, which provides nutrients for the development of the embryo while the VgR is a receptor located on the oocyte surface that is essential for vitellogenin endocytosis. Relatively low levels of Vg2 in egg-laying mites from both control and varnished mites is probably a result of recent oviposition. Decrease in Vg transcription post oviposition was shown before by Cabrera Cordon et al. [20].

## 5. Conclusions

The presented data clearly indicate the crucial role of the foreleg pit organ in the *Varroa*–honeybee interactions and thus in its survival. Other chemosensory organs if they exist apparently play minor role, if any, in honeybee recognition by the *Varroa* mite. Taken together, these data further support the idea to develop future *Varroa* management based on chemosensory disrupting chemicals.

## Supplementary Materials

The following are available online at https://www.mdpi.com/2075-4450/11/6/395/s1, Table S1: List of primers used for qPCR in the present study. Video S1: The effect of foreleg varnishing on mobility and ability of Varroa mite to find and feed on nurse honeybee
